# Hypercalcemia in Cancer: Causes, Effects, and Treatment Strategies

**DOI:** 10.3390/cells13121051

**Published:** 2024-06-18

**Authors:** Patrycja Bartkiewicz, Dominika Kunachowicz, Michał Filipski, Agata Stebel, Julia Ligoda, Nina Rembiałkowska

**Affiliations:** 1 Faculty of Medicine, Wroclaw Medical University, Pasteura 1, 50-367 Wroclaw, Poland; patrycja.bartkiewicz@student.umw.edu.pl (P.B.); michal.filipski@student.umw.edu.pl (M.F.); agata.stebel@student.umw.edu.pl (A.S.); julia.ligoda@student.umw.edu.pl (J.L.); 2 Department of Pharmaceutical Biochemistry, Faculty of Pharmacy, Wroclaw Medical University, Borowska 211 A, 50-556 Wroclaw, Poland; dominika.kunachowicz@student.umw.edu.pl; 3 Department of Molecular and Cellular Biology, Faculty of Pharmacy, Wroclaw Medical University, Borowska 211 A, 50-556 Wroclaw, Poland

**Keywords:** hypercalcemia, intracellular calcium signaling, tumor-induced hypercalcemia, calcium-sensing receptor, parathyroid hormone-related peptide, PTHrP and CaSR receptors

## Abstract

Calcium plays central roles in numerous biological processes, thereby, its levels in the blood are under strict control to maintain homeostatic balance and enable the proper functioning of living organisms. The regulatory mechanisms ensuring this balance can be affected by pathologies such as cancer, and as a result, hyper- or hypocalcemia can occur. These states, characterized by elevated or decreased calcium blood levels, respectively, have a significant effect on general homeostasis. This article focuses on a particular form of calcium metabolism disorder, which is hypercalcemia in neoplasms. It also constitutes a summary of the current knowledge regarding the diagnosis of hypercalcemia and its management. Hypercalcemia of malignancy is estimated to affect over 40% of cancer patients and can be associated with both solid and blood cancers. Elevated calcium levels can be an indicator of developing cancer. The main mechanism of hypercalcemia development in tumors appears to be excessive production of parathyroid hormone-related peptides. Among the known treatment methods, bisphosphonates, calcitonin, steroids, and denosumab should be mentioned, but ongoing research promotes progress in pharmacotherapy. Given the rising global cancer prevalence, the problem of hypercalcemia is of high importance and requires attention.

## 1. Introduction

Calcium is essential for maintaining physiological balance in organisms, regulating various vital functions crucial for homeostasis. Its levels are tightly controlled by hormonal systems like parathyroid hormone and calcitonin, ensuring optimal concentrations in the blood. Calcium serves as a critical second messenger in cells, regulating key biological processes such as cell cycle, differentiation, migration, and cell death. Additionally, it plays roles in muscle contraction, cell membrane stabilization, enzyme activation, and blood clotting. Imbalances in calcium homeostasis can lead to disorders like osteoporosis and complications associated with tumors, including hypercalcemia. Understanding calcium’s role is vital for maintaining health, particularly in bone homeostasis, muscle function, nervous system activity, and blood clotting. Hormonal mechanisms ensure adequate calcium balance, but abnormalities, such as in cancer or metabolic disorders, can lead to hypercalcemia or hypocalcemia, impacting overall health. This review focuses on hypercalcemia, exploring its effects on biological functions and its association with diseases, especially tumors.

## 2. Definition of the Problem

Hypercalcemia is a medical condition defined as an increase in the serum calcium level in blood above the upper limit of the reference range (>10.4 mg/dL). The causes of that medical condition are various, including overactive parathyroid glands, vitamin D disorders, or medications, but most frequently the underlying reasons are primary hyperparathyroidism and cancer, which is the most common cause of hypercalcemia in hospitalized patients [1,2]. Patients with cancer are commonly afflicted with hypercalcemia of malignancy (HCM) according to research, it can affect up to 44% of patients. The motive is that cancer can disrupt the balance between bone formation and resorption through systemic factors released by the primary tumor and local effectors produced by metastasized tumor cells [3]. The sufferers present characteristic symptoms that can include failure to thrive, poor feeding, constipation, and polyuria, making it an oncologic emergency that requires urgent treatment, because usually hypercalcemia observed in advanced cancers is associated with poor prognosis [2].

To understand the biological bases of hypercalcemia in malignant tumor changes, it is worth mentioning the regulatory mechanisms that affect calcium homeostasis. Calcium levels are influenced by parathyroid hormone (PTH), 1,25-dihydroxyvitamin D (1,25[OH]_2_D), and to a lesser extent calcitonin [4,5]. The concentrations of calcium and phosphorus ions remain interrelated, in which the basic regulation mechanism is PTH. Under the influence of calcium levels falling below 10 mg/dL, PTH secretion occurs, which interacts with metabolic transformations of the bones and increases calcium absorption in the further part of the nephron while simultaneously reducing phosphorus absorption in the kidneys [3,4,5]. Through 1-α-hydroxylase, PTH causes hydroxylation of 25-hydroxyvitamin D (25[OH]D) to its active form (1,25[OH]_2_D). This transformation affects the increased absorption of calcium and phosphorus in the intestines [5]. Calcitonin plays a lesser role in calcium homeostasis by decreasing the reabsorption of both calcium and phosphorus, yet it remains an important point of focus for therapy in hypercalcemia of malignancy [3,4]. Additionally, the hormone fibroblast growth factor 23 (FGF23), which under the influence of high phosphate levels stimulates the increased excretion of phosphorus through the kidneys and decreases the level of 1,25[OH]_2_D by reducing the expression of 1-α-hydroxylase, indirectly affects calcemia [6]. In cancer diseases, these numerous regulatory mechanisms are disrupted, which can result in a dangerous complication such as hypercalcemia.

Multiple myeloma is characterized by the highest occurrence of hypercalcemia among both solid and blood cancers, with breast cancer at number two, followed by renal cancer and squamous carcinoma originating from any tissue. Hypercalcemia is also relatively often associated with leukemia and non-Hodgkin lymphoma [7].

Although various mechanisms, including calcium homeostasis disruption which often occurs in tumors, can contribute to the development of cancer-related hypercalcemia, increased bone resorption is indicated as a major etiological factor, particularly in patients with bone metastases [8]. Bones belong to the most common metastatic sites of advanced cancers due to their unique microenvironment formed by osteoclasts, osteoblasts, bone stromal cells, and fenestrated capillaries [9]. Also, the high level of mineralization and diversity of cell populations residing in the bone marrow increases its value as a metastatic niche for tumor cells [10]. The molecular interactions between cancer cells and the bone microenvironment, in some cases referred to as immunosuppressive, form a so-called vicious cycle that aggravates tumor progression and malignancy. In this cycle, metastatic tumor cells settled in the bone marrow release osteolysis-activating factors, and tumor growth is simultaneously stimulated by growth factors from resorbed bone [11,12].

Calcium besides its essential functions of regulating muscle contraction via interaction with troponin and calmodulin and maintaining the structure of bones, plays a variety of other physiological roles, e.g., in stabilization of cell membranes, activation of enzymes, or participation in coagulation cascade [5]. The ionized, active form of calcium (Ca^2+^) acts as a ubiquitous second messenger, which is fundamental in processes involved in cell cycle control, differentiation, cell migration, and angiogenesis, as well as cell death mechanisms. These are closely related to cancer onset, progression, and invasiveness [13,14].

Formation of bone metastases is associated with alterations in bone metabolism, with key importance of parathyroid hormone-related protein and calcium-sensing receptors (CaSRs) at the molecular level [15]. Because the majority of the calcium pool is stored in bones, tumor bone metastases formation including processes of enhanced bone resorption and osteolytic lesions formation, can lead to the release of excessive amounts of calcium into the bloodstream [16]. The following deregulation of calcium homeostasis entails multiple clinical manifestations as it affects the functionality of numerous organs and systems, from gastrointestinal or muscular to the nervous system in the case of more severe hypercalcemia, and its symptoms can range from mild to even occasionally life-threatening [17].

### 2.1. Causes

When discussing the causes of HCM, we can distinguish four basic mechanisms leading to an increase in calcium concentration in the blood. The most common form (approximately 80% of all cases) is humoral hypercalcemia caused by the production of parathyroid hormone-related peptide (PTHrP). The next most frequent cause is bone metastases (osteolytic hypercalcemia), which account for approximately 20% of hypercalcemia in cancer. Excessive production and activation of extrarenal 1,25-dihydroxyvitamin D is responsible for 1% of cases, while the rarest cause, accounting for less than 1% of all cancer-related hypercalcemia, is ectopic secretion of parathyroid hormone [5,7,18]. Generally, hypercalcemia in cancer is most often caused by increased bone resorption and insufficient ability of the kidneys to eliminate excess amounts of calcium [7].

**Figure 1:**
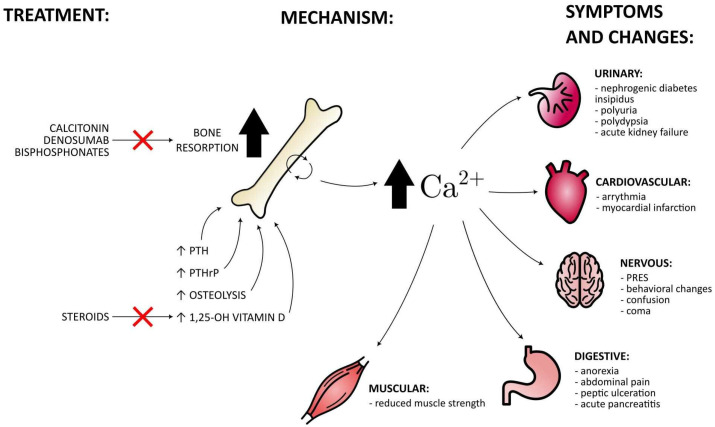
The diagrammatic representation showcases the impact of the hypercalcemia mechanism on the human body and its characteristic symptoms.

It should be noted that the frequency of particular etiological factors of hypercalcemia caused by malignancy is controversial. A cohort study published in 2021 presents a different distribution of the etiology of the described issue. Analyzing the mechanisms responsible for hypercalcemia in all cancer types, PTHrP was present in 27% of cases, while osteolytic metastases were observed in 50%, 1,25-OH vitamin D was associated with approximately 39% of all cases (Figure 1). The discrepancies mentioned previously may be due to incomplete assessment of patients or to changes in the incidence of particular causes over the years [19].

#### 2.1.1. Humoral Hypercalcemia of Malignancy

The issue of this type of hypercalcemia along with a detailed description of PTHrP functions will be discussed later in this work.

#### 2.1.2. Bone Metastases (Osteolytic Hypercalcemia)

This type of hypercalcemia occurs primarily in multiple myeloma, breast cancer, and (much less frequently) in leukemias and lymphomas. The mechanism underlying osteolytic hypercalcemia is indirect bone destruction by metastatic cancer cells that produce substances that influence the formation of osteoclasts [20]. The basic factors determining the bone destruction process are interleukins (IL-1, IL-3, IL-6), prostaglandin E, transforming growth factor α and β (TGFα, TGFβ), lymphotoxin and tumor necrosis factor α (TNFα) [7]. These cytokines stimulate osteoblasts to produce RANKL and activate osteoclasts, leading to bone destruction and the release of calcium into the body [5]. High osteoclast activity increases bone metastasis and metastatic growth, whereas blocking osteoclast activity reduces both osteolytic and osteosclerotic metastases [21].

A noteworthy fact is that metastatic breast cancer cells can additionally produce PTHrP locally, without significant changes in serum PTHrP concentrations [3]. In the case of multiple myeloma and some types of lymphoma, macrophage inflammatory protein-1α (MIP1α) plays an important role, as it stimulates osteoclastogenesis and inhibits the process of differentiation of stromal cells into osteoblasts. This happens when cancer cells infiltrate the bone marrow. An additional cause of the increase in calcium levels is paraproteins produced in the described cancer processes, which additionally bind calcium [7,20].

#### 2.1.3. Excessive Production and Activation of Extrarenal 1,25-Dihydroxyvitamin D

The mechanism of hypercalcemia based on excessive extrarenal production of 1,25[OH]_2_D concerns primarily lymphomas (non-Hodgkin and Hodgkin), ovarian dysgerminoma and appears in other, non-malignant processes (e.g., tuberculosis and sarcoidosis) [7,20]. In Hodgkin lymphomas, it is the main cause of hypercalcemia, while in non-Hodgkin lymphomas, it is responsible for ⅓ of all cases. It should be noted that the main cause of hypercalcemia in non-Hodgkin lymphomas is not related to PTHrP or 1,25-dihydroxyvitamin D and remains unknown [20,22]. It has also been shown that in the course of cancer hypercalcemia, increased 1,25-dihydroxyvitamin D concentration is associated with an increased risk of incomplete response to antiresorptive treatment in patients with solid tumors [22]. Moreover, hypercalcemia accompanied by high 1,25[OH]_2_D levels seems to be a marker of more aggressive and treatment-resistant lymphomas [23].

In a healthy body, the production of 1,25[OH]_2_D by the enzyme 1α-hydroxylase is regulated by the parathyroid hormone, but in hypercalcemia associated with cancer, this regulation is significantly impaired. Cancer cells in the surrounding tissue macrophages excessively express 1α-hydroxylase, leading to excessive conversion of 25-hydroxyvitamin D into 1,25[OH]_2_D. The product of this pathologically intensified process causes excessive calcium absorption in the intestines and leads to hypercalcemia [7].

#### 2.1.4. Ectopic Secretion of Parathyroid Hormone

Elevated PTH concentration is an extremely rare cause of hypercalcemia in malignant tumors. This phenomenon may occur in the case of neuroendocrine tumors, pancreatic carcinoma, ovarian carcinoma, lung carcinoma, neuroectodermal tumor, rhabdomyosarcoma, thyroid papillary and medullary carcinoma, and gastric carcinoma. In this case, cancer cells ectopically produce PTH [7,24,25]. When discussing the topic of parathyroid hormone, it should be noted that primary hyperparathyroidism caused by parathyroid hyperplasia or parathyroid adenoma is also possible in oncological patients [4].

### 2.2. Symptoms and Changes in Individual Organs

#### 2.2.1. Digestive System

The digestive system, through the CaSR, actively participates in the regulation of calcium homeostasis by controlling its intake and efflux. CaSR expression is ubiquitous across all digestive organs, exerting a pivotal influence on systemic calcium balance [26]. In the stomach, receptor activation stimulates the release of gastrin, enhancing hydrochloric acid (HCl) production to facilitate efficient calcium absorption. Deactivation of CaSR transpires in response to elevated serum calcium levels. Sustaining an elevated intracellular Ca^2+^ concentration supports HCl production by promoting pump translocation [27]. Furthermore, the CaSR receptor is present in the small and large intestines, where it may play a role in regulating bowel movements [28].

Hypercalcemia within the digestive system manifests with symptoms such as anorexia, constipation, cramping abdominal pain, and peptic ulceration. When hypercalcemia reaches high levels, it may lead to nausea and vomiting, and in severe cases, acute pancreatitis [1,3,8]. Elevated levels of calcium inhibit neuromuscular depolarization, contributing to decreased smooth muscle tone and consequent autonomic dysfunction. Pancreatitis ensues through the abnormal accumulation of intracellular calcium ions, leading to the excessive activation of trypsinogen and resulting in pancreatic autodigestive injury [29,30].

Recent findings indicate that some patients with significant hypercalcemia experience dysphagia [31,32], which is a dysfunction characterized by difficulty in effectively moving food from the mouth to the stomach. Dysphagia may occur in patients with significant hypercalcemia, where there is an excessively high level of calcium in the blood. The impact of elevated calcium concentrations on the muscles and nervous system, which are responsible for regulating the swallowing process, may be an important factor contributing to this phenomenon [31].

#### 2.2.2. Urinary System

Severe hypercalcemia can induce renal dysfunction through multiple molecular pathways, encompassing aberrations in transcellular ion transport and diminished renal blood flow. Deposition of calcium in the medulla and downregulation of aquaporin 2 (AQP2) channels in collecting ducts result in dysregulated urine concentration that can be demonstrated in patients as polyuria and dehydration [5]. Additionally, the reabsorption of calcium and sodium chloride in the ascending limb of the loop of Henle is reduced. That is the result of CaSR activation, combined with inhibition of sodium/potassium/chloride cotransporter and production of prostaglandin E2 [33]. On the other hand, reduced volume activates the renin–angiotensin–aldosterone system, which stimulates the retention of sodium and reabsorption of calcium in the proximal renal tubule. Decreased glomerular filtration rate (GFR) and poor renal blood flow are thought to be consequences of direct renal afferent arteriole vasoconstriction [5,33].

The most important outcome of these molecular mechanisms is excessive water loss. The clinical manifestation of hypercalcemia in the urinary system is nephrogenic diabetes insipidus, which results in polyuria and polydipsia. Moreover, patients can develop conditions such as distal renal tubular acidosis [3] and acute kidney failure (AKI). Fortunately, AKI caused by hypercalcemia is usually reversible when properly managed [33]. Renal effects of chronic hypercalcemia may include nephrolithiasis, tubular dysfunction, and chronic renal failure [3].

#### 2.2.3. Nervous System

Calcium ions play a pivotal role in various neurobiological phenomena, encompassing synaptic transmission, cognitive processes, and consolidation of memory [34]. Additionally, these ions regulate the enduring enhancement of synaptic transmission known as long-term potentiation (LTP) induced by high-frequency stimulation. This regulatory role influences the modification of synaptic plasticity by synapses. The direct interplay between the depolarization of the plasma membrane and the elevation of intracellular Ca^2+^ signifies a set of specific neuronal processes, all meticulously orchestrated by calcium signals [35]. Patients experiencing mild hypercalcemia may demonstrate behavioral changes, such as heightened anxiety, depression, mood fluctuations, and a decline in cognitive function. Conversely, individuals with severe hypercalcemia may display symptoms such as myopathy, confusion, delirium, and coma [1,3].

In cases of hypercalcemia associated with malignancy, patients may develop a syndrome known as posterior reversible leukoencephalopathy (PRES) which is typified by a clinical and radiological profile featuring white matter vasogenic edema predominantly affecting the posterior occipital and parietal lobes of the brain [36]. While hypertension is the primary causative factor, hypercalcemia can also contribute to the development of PRES [3]. PRES can present with a range of manifestations, spanning from acute to subacute, including seizures, impaired visual acuity, visual field deficits, disorders of consciousness, headaches, confusion, and focal neurological deficits [8].

#### 2.2.4. Cardiovascular System

Increased calcium levels in the blood also have a significant impact on the cardiovascular system. The symptoms of hypercalcemia vary depending on the level of calcium in the blood. In the case of severe hypercalcemia (corrected calcium concentration >14 mg/dL), we may experience cardiac arrest, arrhythmia, and ventricular tachycardia. Moderate and mild hypercalcemia (corrected calcium levels of 12–13.9 mg/dL and 10.5–11.9 mg/dL) may be manifested by the following ECG changes: ST-segment depression, shortening of the QT interval, and prolongation of the PR and QRS intervals [37]. Calcium has a positive inotropic effect up to a concentration of approximately 15 mg/dL, from which point it has a depressing effect on the strength of myocardial contraction. Moreover, many patients with hypercalcemia develop secondary hypokalemia, which increases the risk of cardiac arrhythmias [38]. Very high concentrations of calcium in the blood may cause conditions that mimic ST-segment elevation myocardial infarction. In such cases, coronary artery catheterization usually does not show any significant changes, and correction of electrolyte disturbances leads to the disappearance of symptoms [39,40].

#### 2.2.5. Muscular System

Since there is a close association between bone and muscle physiology, bone metastases due to increased bone resorption, disruption of the balance between osteoblasts and osteoclasts activity, and alterations in various signaling pathways can induce changes in muscle function. Similarly, factors released in response to muscle cells can aggravate bone resorption processes, and thereby exacerbate muscular dysfunction [41]. Bone resorption is accompanied by the release of proinflammatory cytokines, which contribute to the dysfunction of muscle cells [42]. Preclinical studies on mouse models confirm that invasive MDA-MB-231 breast cancer, A549 lung cancer, PC3 prostate cancer, and JJN3 multiple myeloma bone metastases impaired muscle function, causing lower muscle strength and muscle-specific force in comparison to control mice [41,43]. It is mediated by TGF-β released from the surface of metastatic bones as a result of their destruction, followed by oxidation of skeletal muscles, sarcoplasmic reticulum calcium leak due to oxidation of ryanodine receptor and calcium release channel (RyR1), and finally impairment of intracellular signaling. Otherwise, in normal conditions, Ca^2+^ stored in the sarcoplasmic reticulum is released as a result of RyR1 activation during excitation–contraction coupling in skeletal muscle, enabling Ca-dependent cross-linking of actin and myosin which results in muscle contraction [44].

PTHrP-induced bone resorption occurs via RANK receptor activation in stromal osteoprogenitors. The following signaling cascade induces calcium release from ER which in consequence reduces its reserve. Furthermore, this ligand-receptor binding enhances the differentiation of osteoclast precursors, their maturation, and in consequence bone remodeling, i.e., osteoclastic bone resorption [45]. Hypercalcemia due to calcium release, accompanying the above-described processes, in the muscular system is manifested mainly by reduced muscle strength, observed both in moderation [46,47]. However, it is worth noting that muscle weakness can be of a central nervous origin, but at the same time, hypercalcemic conditions increase the neuromuscular excitation level. Moreover, hypokalemia contributes to reduced muscle strength [48].

## 3. Markers and Diagnosis

The diagnosis of hypercalcemia can be misleading. The most frequently studied parameter is the concentration of total calcium, which includes both the protein-bound and free forms. It should be noted that only the unbound or ionized form is biologically active. The vast majority of calcium is bound to albumin; therefore, in patients with abnormal albumin levels, calcium measurement should be corrected according to the following formula: total serum calcium concentration + 0.8 × (4 − albumin concentration). The result of this formula is defined as the corrected calcium concentration (CRC). Unfortunately, the described methods have their limitations because albumin is not the only carrier of calcium, which becomes more important when cancer cells produce paraproteins that can bind calcium (especially important in the case of multiple myeloma). Therefore, the preferred method is to determine the concentration of ionized calcium, which is more sensitive than the previously described tests [7,46]. Unfortunately, this parameter also has its limitations because it depends on the blood pH (lowering the pH results in an increase in the concentration of calcium ions). Of course, calcium levels should be repeated to confirm the diagnosis [4].

In the diagnosis of hypercalcemia, apart from laboratory tests, an extremely important issue is the assessment of the patient’s clinical condition and a carefully conducted medical interview. An accidentally detected elevated calcium level may turn out to be the first symptom of cancer. The patient should be asked about recently performed screening tests (appropriate to the patient’s gender and age), the presence of a cough, increased weight loss, or other disturbing changes. The history of previous diseases and family history of diseases (in particular, hyperparathyroidism, cancer, kidney stones) may be important. The interview should take into account the medications taken since vitamin A and D supplementation, lithium, and thiazide diuretics may disturb calcium homeostasis. It is reasonable to ask about smoking, alcohol consumption, and other carcinogenic factors. During a physical examination, it is worth paying special attention to the symptoms of hypovolemia [16].

The most common causes of hypercalcemia in clinical practice are primary hyperparathyroidism and cancer. It is worth noting here that cancer patients usually have higher calcium levels than people with secondary hypercalcemia and the symptoms of this disorder are more severe in the group of oncology patients [3,49]. Additionally, in the group of patients suffering from HCM, when elevated calcium levels are discovered, clinical symptoms of cancer are usually present [20].

The next diagnostic step after diagnosing hypercalcemia is to identify the mechanism of this disorder. For this purpose, a panel of laboratory tests should be performed, which includes the assessment of PTH, PTHrP, 25[OH]D, 1,25[OH]_2_D, and phosphorus [7].

Humoral hypercalcemia of malignancy should be suspected, especially in patients with solid tumors [50]. In laboratory tests, this mechanism is characterized by an increased PTHrP concentration and a noticeably reduced, or possibly low normal, PTH level in the serum. The diagnosis is confirmed by low concentrations of 1,25[OH]_2_D and phosphorus [7]. Hypercalcemia based on an osteolytic mechanism should be suspected primarily in patients with multiple myeloma as well as metastases infiltrating the bone marrow and bones. Laboratory tests of the serum show increased levels of phosphorus and low concentrations of 1,25[OH]_2_D and PTH [7,50]. Hypercalcemia caused by excessive production and activation of extrarenal 1,25[OH]_2_D occurs especially in patients suffering from lymphoma. In this case, the concentration of 1,25[OH]_2_D in the serum is increased without an increase in the level of 25-hydroxyvitamin D, and the concentration of PTH is reduced [7,50].

The finding of an elevated PTH level leads to the suspicion of hypercalcemia caused by primary hyperparathyroidism, which is more common in oncology patients than in the general population. To confirm this diagnosis, imaging tests can be performed to assess the function of the parathyroid glands, which will help exclude ectopic secretion of parathyroid hormone by cancer cells; this phenomenon is extremely rare [3,7]. Additional tests may also be helpful in the diagnostic process. In cases of suspected hypercalcemia caused by bone metastases, imaging studies such as bone scintigraphy, X-ray, and laboratory tests, including serum and urine protein electrophoresis and free immunoglobulin light chains in serum assay, can be used to confirm the etiology. These tests are helpful diagnostic tools for determining monoclonal gammapathies [7]. It is worth mentioning that determining the level of vitamin A and TSH may be helpful in the diagnosis of hypercalcemia [51].

### 3.1. The Role of Parathyroid Hormone-Related Protein

Parathyroid hormone-related peptide (PTHrP) is a hormone that has structural similarity to parathormone, especially to its N-terminal sequence. Due to this resemblance, PTHrP can bind to the type 1 PTH receptor and have some PTH biological effects [16,52]. PTHrP is produced in various tissues in very low amounts as a propeptide, afterwards, it is cleaved by prohormone convertase to a mature form [51].

Depending on the source, PTHrP can be divided into three or more main parts, which differ in biological role [51]. The first region is the N-terminal domain, which can bind to PTHR1, G-protein-coupled receptor, and in that way activates protein kinase A (via cyclic adenosine monophosphate) or protein kinase C (via phospholipase C and calcium ions) [53]. PTHrP can mimic some of the PTH biological functions by affecting this receptor. The mid-region probably takes part in placental calcium ions transport [51] and facilitates lung healing [54]. The last part of PTHrP, the C-terminal region, is thought to inhibit osteoclast-mediated bone resorption. Some authors indicate that sequences of PTHrP that direct this peptide to the nuclear site of a cell (nuclear localization signal) may be seen as a separate region [51,53], although it should be considered that the division of this hormone in parts is historical and simplified to understand the structure and function of PTHrP.

PTHrP can be found in humans in three isoforms containing 139, 141, or 173 amino acids, in contrast to other mammals in which only one splice variant is present. Transcription of each isoform is regulated by different promoters [53,54]. Secretion of PTHrP is regulated on the level of gene transcription. While many cytokines and growth factors stimulate that process (e.g., epidermal growth factor, EGF, IGF-1, and transforming growth factor-β,TGFβ), steroidal hormones like 1,25[OH]_2_D, glucocorticoids, and androgens seem to be inhibitors of transcription [51].

PTHrP normally influences cells locally by autocrine, paracrine, or intracrine effect, while long-range, humoral mechanisms occur rarely. There are three known cases in which PTHrP acts rather as an endocrine factor: lactation, fetal development, and last but not least, HHM [53,54].

#### 3.1.1. Role of Parathyroid Hormone-Related Peptide (PTHrP) in a Healthy Organism

The main known functions of PTHrP in normal cells are associated with pregnancy and the production of breast milk. In fetus tissues, PTHrP plays a vital role in calcium homeostasis and takes part in embryogenesis by facilitating the transport of calcium through the placenta [3,53]. Based on animal models we can assume that mid-region, especially with 67–86 and 38–94 amino acids, is involved in that process, and it is necessary for proper mineralization of the fetal skeleton [54]. In mammary glands, circulating PTHrP enhances transferring calcium ions to the breast milk. Binding to PTH1 on osteoblasts and activation of osteoclasts leads to the release of excess amounts of bone calcium, which is subsequently used for breast milk production [51].

PTHrP influences the development of tissues by regulating proliferation, differentiation, and programmed cell death. This can be observed in the breast, both in normal tissues and neoplasms [55], skin, nervous system, and pancreas, but most importantly in bones. Normal development of the cartilaginous growth plate and endochondral bone formation requires the activity of PTHrP, which is secreted in the distal perichondrium and it controls maturing of chondrocytes via paracrine PTHrP/Indian hedgehog signaling loop [53,56]. PTHrP is also known to cause smooth muscle relaxation in vessels, the urinary bladder, uterus, stomach, and intestine. Its local production is rapidly stimulated by vasoconstrictors like angiotensin II, which may suggest that PTHrP secretion may function as a mechanism regulating or limiting smooth muscle constriction [51,54].

#### 3.1.2. Role of Parathyroid Hormone-Related Peptide (PTHrP) in Hypercalcemia

PTHrP increases the reabsorption of calcium [46,51] and excretion of phosphorus in the kidneys, which results in high calcium levels in serum with simultaneous hypophosphatemia [4]. PTHrP also causes elevated urinary cyclic AMP excretion [52]. Besides the effect on kidneys, PTHrP can influence calcium homeostasis by its metabolic impact on bones. In bone-metastatic tumors and multiple myeloma, PTHrP is produced locally without an overall increase in serum level. By binding to the PTHR1 receptor on osteoblasts, PTHrP enhances the release of RANKL (receptor activator of factor κB ligand). RANKL then binds to the RANK receptor (Receptor Activator for Nuclear Factor κB) on osteoclasts. This molecular interaction intensifies the maturation of osteoclasts, which results in bone resorption and elevates calcium concentration [3,16]. The process of bone resorption causes the secretion of growth factors such as transforming growth factor beta (TGF-β), bone morphogenetic protein (BMP), and insulin-like growth factor (IGF) 1 and 2, which stimulate the growth of the metastatic tumor. Moreover, TGF-β via activation of SMAD (suppressor of mothers against decapentaplegic) complexes and GLI2 (transcription factor Gli2) increase PTHrP transcription. This mechanism is known as a vicious cycle of bone destruction [53].

PTHrP, in contrast to PTH, does not influence the 1,25-dihydroxycholecalciferol production [51,56]; therefore, it does not affect intestinal absorption of calcium and phosphorus [4]. However, cases of hypercalcemia with simultaneous overproduction of PTHrP and 25(OH)D have been occasionally reported [57]. Hypercalcemia linked to humoral or local overproduction of PTHrP usually occurs in neoplasms. There are examples of hypercalcemia caused by overproduction of PTHrP in the placenta or mammary glands during pregnancy, puerperium, or lactation; nonetheless, this condition is rather rare [58].

#### 3.1.3. Role of Parathyroid Hormone-Related Peptide (PTHrP) in Neoplasms

Biological effects of PTHrP are especially important in neoplasms although excessive synthesis of PTHrP is considered to be the most common mechanism in hypercalcemia related to malignancy. Ectopic PTHrP production by tumor cells is called humoral hypercalcemia of malignancy (HHM) [16,46] and according to certain sources, it is estimated that 80% of cases of hypercalcemia in neoplasms develop based on this mechanism [3,56]. Particular types of malignancies are associated with systemic secretion of PTHrP, for example, tumors like cell carcinomas of the lung, head and neck, esophagus, skin, or cervix, carcinomas of the breast, kidney, prostate, and bladder cancer of the ovary and non-Hodgkin lymphoma [3,16]. Studies show that among malignancies with hypercalcemia mediated by the production of PTHrP, approximately 85% of cases are solid organ malignancies, and the remaining 15% are mainly hematological malignancies. Unfortunately, hypercalcemia developing through excessive secretion of PTHrP is a poor prognostic factor, patients diagnosed with that condition have a median overall survival of less than 2 months [56]. It is worth mentioning that PTHrP can be overproduced only locally without an increase in the serum level of PTHrP. This can occur in the metastasis of breast cancer to bones, where PTHrP production is stimulated by the secretion of TGF β and other factors [3].

#### 3.1.4. Role of Parathyroid Hormone-Related Peptide (PTHrP) in Diagnostic Process

The level of PTHrP can be a useful tool in establishing the origin of hypercalcemia. Considering the most common cause of hypercalcemia, which is primary hyperparathyroidism (PHPT), the first step in the diagnostic process should be excluding this condition, by measuring the amount of PTH. High levels of PTH usually indicate PHPT or, less frequently, parathyroid cancer. Patients suffering from hypercalcemia with low PTH are most likely to have hypercalcemia of malignancy (HCM); therefore, the level of PTHrP should be checked to confirm the presence of humoral hypercalcemia of malignancy. In formerly undiagnosed patients, the underlying malignancy should be investigated [16]. PTHrP concentration is high in HCM, but it can also be elevated during breastfeeding and among postmenopausal women with low body mass index [56].

The most commonly used PTHrP assay detects the C-terminal of this hormone regarding the fact that levels of circulating C-terminal PTHrP are much higher than N-terminal PTHrP. The main weakness of this survey is the fact that C-terminal PTHrP accumulates in patients with renal failure, which may falsify the outcome, showing elevated PTHrP. This is why in peculiar cases of renal diseases, N-terminal PTHrP assay should be performed as the result of this test, which is not influenced by low GFR [56].

### 3.2. The Role of Calcium-Sensing Receptors

The calcium-sensing receptors (CaSRs) belong to the family C of the superfamily G-protein-coupled receptors. The main role of this receptor is to maintain calcium ion homeostasis. It is expressed at high levels in the chief cells of the parathyroid glands, the kidneys, the gastrointestinal tract, and bones—the most important organs of this homeostasis [59,60]. Furthermore, its significance extends to various tissues, including the thyroid, breasts, colon crypt cells, keratinocytes, fibroblasts, ovarian surface cells, blood vessels, brain, liver, and placenta [61]. The CaSR is a physiologically significant receptor that plays a crucial role in the regulation of digestion, absorption, appetite, and whole-body metabolism (Table 1). Additionally, it actively participates in essential cellular processes such as proliferation, differentiation, and apoptosis. Dysregulation of these processes may directly contribute to the initiation and progression of tumorigenesis [62].

CaSR is expressed in cells that serve as targets for PTH and calcitonin (CT) in the kidneys, bones, and intestines. It actively participates in “short-loop” feedback systems, enabling the detection of local changes in Ca^2+^ concentrations within tissues and thereby facilitating the maintenance of homeostasis. Additionally, CaSR serves as a sensor for pH. Alterations in pH within the range from 6 to 8.5 modulate the receptor’s sensitivity to the concentrations of calcium and magnesium ions. This holds paramount importance in physiology and the regulation of pH within bone cells and the gastrointestinal tract [61].

#### 3.2.1. Parathyroid Gland

Elevated levels of Ca^2+^ and the subsequent activation of CaSR also increase the expression of CaSR and vitamin D receptor genes. Vitamin D receptors participate in the reduction in PTH gene expression. Additionally, CaSR is involved in the intracellular degradation of PTH during hypercalcemia. The functionality of the parathyroid gland is contingent upon the concentration of Ca^2+^ in the bloodstream. The CaSR acts as a receptor responsive to this concentration, assuming a pivotal role in preserving Ca^2+^ homeostasis through the regulation of processes such as PTH secretion, PTH gene expression, and parathyroid cellular proliferation. Hypocalcemia serves to stimulate these processes, while hypercalcemia exerts inhibitory effects. Elevated levels of Ca^2+^ and the consequent activation of CaSR further elevate the expression of both CaSR and vitamin D receptor genes. Vitamin D receptors actively contribute to the reduction in PTH gene expression. Furthermore, CaSR is intricately involved in the intracellular degradation of PTH during episodes of hypercalcemia [63].

#### 3.2.2. Thyroid

The role of CaSR in the thyroid is primarily associated with regulating blood calcium levels through the secretion of calcitonin. Calcitonin is secreted by parafollicular C-cells, which express CaSRs. Hypercalcemia stimulates CaSRs on parafollicular C-cells, leading to the secretion of calcionin. The main function of this hormone is to lower blood calcium levels by inhibiting calcium resorption from bones and reducing its absorption from the gastrointestinal tract. Therefore, CaSRs in the thyroid play a significant role in regulating the body’s calcium homeostasis [64].

#### 3.2.3. Bones

The CaSRs are expressed in the principal bone cells, osteoblasts, and osteoclasts. Calcium is a pivotal element in bone turnover and Ca^2+^ homeostasis. The proliferation of osteoblasts is stimulated by Ca^2+^, while that of osteoclasts is inhibited. CaSR regulates growth plate chondrogenesis and stimulates longitudinal bone growth [50].

#### 3.2.4. Gastrointestinal Tract

CaSR is expressed in all organs of the digestive system. Increased intake of dietary calcium stimulates the activation of CaSR. In the esophagus, CaSR is expressed in the basal cells and the non-tumorigenic esophageal epithelial cell line HET-1A. CaSR may play a role in the proliferative response to injury and the pathogenesis of esophagitis. Currently, it remains uncertain whether CaSR influences the proliferation and differentiation of cancer cells in esophageal cancer.

In the liver, CaSR is essential for maintaining physiological functions. During hepatic ischemia/reperfusion, the activation of CaSR influences cell apoptosis. In cirrhosis, CaSR reduces intrahepatic resistance to portal flow. In the pancreas, CaSR monitors and regulates the concentration of calcium ions in pancreatic juice by triggering ductal electrolyte and fluid secretion, consequently reducing the precipitation of calcium ion salts in the duct lumen, thereby decreasing the risk of carbonate stone formation. Additionally, CaSR participates in the regulation of glucose-induced insulin secretion by pancreatic β-cells.

In the stomach, calcium ions stimulate HCl secretion, and the presence of CaSR in the stomach contributes to the regulation of HCl secretion. In the intestines, CaSR is present in colonic crypts, playing a role in the regulation of normal intestinal epithelial cell proliferation and differentiation. CaSR is involved in the regulation of water and calcium ion absorption [26].

#### 3.2.5. Kidneys

The kidneys play a crucial role in calcium homeostasis. CaSR is expressed nearly along the entire length of the nephron, undertaking three distinct functions: firstly, it inhibits the inhibitory effect of PTH on renal phosphate reabsorption in the proximal tubule; secondly, it inhibits renal calcium excretion in the cortical thick ascending limb (cTAL) of the loop of Henle; and thirdly, it diminishes the urinary concentrating capacity in the inner medullary collecting duct [65].

#### 3.2.6. Brain

CaSR is additionally situated within the subfornical organ of the third ventricle in the brain. This anatomical locale assumes a pivotal role in regulating thirst perception. Consequently, individuals afflicted with hypercalcemia often experience heightened sensations of thirst. Additionally, the CaSR plays a significant role in processes such as memory formation, cognition, and motor reflexes [66,67].

#### 3.2.7. Breasts

Research suggests that CaSRs may play a role in breast cancer development and progression. Activation of CaSRs has been linked to the promotion of cell proliferation, migration, and invasion in breast cancer cells. Additionally, CaSRs may influence the response of breast cancer cells to therapies targeting calcium signaling pathways [68].

In lactating women, CaSRs play a crucial role in regulating calcium homeostasis and milk production. These receptors are expressed in the epithelial cells of the mammary glands, where they facilitate the transfer of calcium from the maternal circulation into the breast milk. By sensing changes in calcium levels, CaSRs help maintain the appropriate concentration of calcium in the milk, which is essential for the growth and development of the nursing infant. Additionally, CaSRs are involved in inhibiting the secretion of PTHrP, which can contribute to calcium mobilization from maternal bones during lactation. Therefore, the expression and function of CaSRs in the mammary glands are critical for ensuring adequate calcium supply to support lactation and infant nutrition [68].

### 3.3. The Role of Calcium-Sensing Receptors (CaSR) in Cancer

In cancer cells, intracellular calcium signaling is commonly deregulated. CaSR may either impede or facilitate tumorigenesis, contingent upon the specific cancer type [60]. It serves as a tumor suppressor in neuroblastoma, parathyroid, gastric, and colon cancer while functioning as an oncogene in ovarian, breast, kidney, and prostate cancer [69].

#### 3.3.1. Colorectal Cancer

In the colon, the CaSR exerts inhibitory effects on proliferation and regulates the terminal differentiation of cells. Consequently, a deficiency of CaSR is evident in colorectal cancer. Its presence is noted in early adenomas and polyps, diminished in advanced adenomas, and absent in the later stages of cancer progression [70]. Furthermore, a study conducted by Iamartino et al. on mice revealed that CaSR participates in the inflammatory response within the intestines. Consequently, its depletion results in diminished integrity of the intestinal barrier and alterations in the composition of the gut microbiota [71].

#### 3.3.2. Parathyroid Cancer

The decline in expression of the CaSR is associated with tumorigenesis in the parathyroid gland. This phenomenon is characterized by diminished control over PTH secretion, as CaSR is responsible for inhibiting PTH release. Moreover, the low or no CaSR expression leads to a lack of control over the differentiation and hyperplasia of parathyroid cells [60,72].

#### 3.3.3. Breast Cancer

In breast cancer, the CaSR acts as a stimulator of tumorigenesis by promoting the secretion of PTHrP, which is responsible for hypercalcemia and cancer progression. Additionally, CaSR is involved in the inhibition of apoptosis and the proliferation of cancer cells. Notably, an observable trend suggests that the elevation of pathological progesterone receptor levels corresponds with increased CaSR expression. Higher CaSR levels correlate with shorter survival times [15].

#### 3.3.4. Prostate Cancer

CaSR exhibits high expression levels within prostate tumors. Correspondingly, heightened expression is correlated with an increased risk of metastasis, especially to bones [73]. A study by Ahearn et al. demonstrated that CaSR may exert influence in promoting vascular growth [74].

## 4. Treatment

The main aim of treatment in the case of hypercalcemia of malignancy is to lower the serum calcium concentration by targeting the underlying disease. It can be achieved by inhibiting bone resorption, increasing urinary calcium excretion, and decreasing intestinal calcium absorption [75]. The type of treatment depends on the severity of hypercalcemia and associated symptoms, for example, asymptomatic hypercalcemia may not need immediate treatment until the diagnosis has been confirmed. But the in case of moderate and severe hypercalcemia, prompt response is required [4]. As shown above, firstly the underlying cancer should be addressed to reduce the production of all the factors that are causing bone resorption and renal calcium reabsorption. In advanced stages of cancer, we can encounter obstacles with the treatment efficacy; therefore, the main purpose will be to inhibit those processes, especially bone resorption [76].

Another problem that needs to be taken into consideration is that patients with hypercalcemia are often dehydrated. The main reason is poor oral intake secondary to nausea, vomiting, altered mental status, and hypercalcemia-induced nephrogenic diabetes insipidus. Therefore, the initial step of the treatment should be rehydration, but realizing it only relieves symptoms and does not substitute the major treatment [3,76]. For that, we can use isotonic crystalloid solutions, such as normal saline to restore renal perfusion. The commonly known furosemide is not recommended due to the development of volume depletion and electrolyte abnormalities [3].

### 4.1. Bisphosphonates as First-Line Therapy

Bisphosphonates are used as the first-line therapy for malignancy-associated hypercalcemia due to their ability to induce osteoclast apoptosis and reduce osteoclastic bone resorption [4]. Their safe and effective way to reduce abnormally high serum calcium levels translates to being the standard of care for treating that condition, but not only. They are useful as well in managing and preventing conditions such as osteoporosis, Paget’s disease of bone, metastatic bone disease, and hypercalcemia [3]. Among them, we find the following synthetic analogs of pyrophosphonate: clodronate, pamidronate, ibandronate, and zoledronic acid, but the preferred ones are pamidronate and zoledronic acid, all administered intravenously. The research shows that zoledronic acid is the one with the greater efficacy compared to pamidronate. It gets a higher proportion of complete responses by day 10, more rapid calcium normalization, and more durable responses [76]. Also, it was revealed that zoledronic acid is almost one thousand times more potent than pamidronate and is more recommended in patients with normal to moderately impaired renal function due to the shorter infusion time [75].

On the other hand, it was also illustrated that patients undergoing the zoledronic acid treatment were more likely to experience side effects, such as osteonecrosis of the jaw, especially in patients who receive high doses for a long time, or renal adverse events [76]. That is the reason why they are reserved for those patients, for whom the benefit rises above the risk, and during the therapy renal function is taken under control, and doses of bisphosphonates are carefully adapted to creatinine clearance GFR [4]. However, they are for now the safest and best-studied class of medicines for treating that condition, and other therapies are used when bisphosphonates prove not sufficient [75].

### 4.2. Calcitonin

Calcitonin is another curative, whose role is particularly important in adjunctive treatment in patients who suffer from severe hypercalcemia due to its rapid action. It is a hormone, which is utilized simultaneously with bisphosphonates and works more efficiently than while taken alone [4,76]. The mechanism relies on the inhibition of osteoclast activity and increasing renal calcium clearance. The research has revealed that a dose of calcitonin is not only important for the effective treatment, but is also a way of administration. It should be given to a patient only intramuscularly or subcutaneously because these ways show expected results. The main side effect that can occur after administration is tachyphylaxis [3,4].

### 4.3. Steroids

Corticosteroids are also used in the treatment of hypercalcemia caused by the disproportionate production of calcitriol. They manage that condition by inhibiting the conversion of calcidiol (25-hydroxyvitamin D) to calcitriol (1,25-dihydroxyvitamin D) which reduces the level of calcium absorber in the intestines [75]. Especially positive results were observed in patients suffering from lymphomas and ovarian germ tumors. The used glucocorticoids are hydrocortisone, with intravenous administration, and prednisone taken orally. If prednisone does not bring the expected results following 10 days of usage, it should be discontinued. Additionally, doctors should be aware of side effects such as hyperglycemia, hypertension, and psychiatric disturbances [3,7].

### 4.4. New Medications

Hypercalcemia of malignancy can be also treated with new therapies such as denosumab, which was approved in some countries, such as the USA, Canada, and Australia. It is a fully human RANKL monoclonal antibody administered subcutaneously that inhibits and reduces the activity of osteoclasts, stopping bone resorption [76]. It was shown that this new medicament could be even more efficacious than zoledronic acid, as well as in reducing the risk of developing recurrent HCM [75]. Unfortunately, in comparison to bisphosphonate, denosumab revealed a higher risk of arthralgias, jaw osteonecrosis, and hypocalcemia, especially at the beginning of the therapy. To reduce hypocalcemia, it is considered to lower the doses and administrate them less frequently [4].

Research is ongoing with an alternative new therapy for HCM, known as cinacalcet. It is a calcimimetic that binds to the CaSR present in parathyroid cells. This interaction results in reducing the production of PTH, which consequently leads to a decrease in serum calcium levels. For this reason, it may be specifically beneficial when hypercalcemia is triggered by malignancy of the parathyroid gland and in secondary hyperparathyroidism [75].

## 5. Conclusions and Future Directions

Hypercalcemia poses significant challenges across multiple bodily systems, affecting digestion, cognitive function, and muscle tone. It often indicates neoplastic activity, necessitating careful monitoring and diagnosis. Treatment typically involves bisphosphonates, calcitonin, steroids, and denosumab, with ongoing research exploring novel therapeutic approaches. Inhibiting excessive parathyroid hormone-related peptide and CaSR activity shows promise for future pharmacotherapy development. Addressing hypercalcemia in cancer patients can improve their quality of life and potentially slow tumor progression, underscoring the importance of continued research in this field.

## Figures and Tables

**Table 1:** The role of CaSR in different human organs and its effects in the selected types of cancers.

Organs or Tracts	The Role of CaSR	Presence of CaSR in Cancer	Effects of Presence or Absence of CaSR in Cancer
Parathyroid Gland	Preserving Ca^2+^ homeostasis through the regulation of processes such as PTH secretion, PTH gene expression, parathyroid cellular proliferation	Low level /absence	Lack of control over the differentiation and hyperplasia of parathyroid cells
Thyroid	Stimulation of CaSRs on parafollicular C-cells leads to secretion of calcitonin	No data	No data
Bones	Regulates growth plate chondrogenesis and stimulates longitudinal bone growth	No data	No data
Colon	The CaSR exerts inhibitory effects on proliferation and regulates the terminal differentiation of cells	Absence	Diminished integrity of the intestinal barrier
Kidneys	Inhibits the inhibitory effect of PTH, inhibits renal calcium excretion in the cTAL of the loop of Henle, diminishes urinary concentration capacity in the inner medullary collecting duct	No data	No data
Breasts	Regulation of calcium homeostasis and milk production	Present	The CaSR acts as a stimulator of tumorigenesis by promoting the secretion of PTHrP, which is responsible for hypercalcemia and cancer progression
